# Distinct Clinical Features and Novel Mutations in Taiwanese Patients With X-Linked Agammaglobulinemia

**DOI:** 10.3389/fimmu.2020.02001

**Published:** 2020-09-04

**Authors:** Yu-Hsin Yeh, Meng-Ying Hsieh, Wen-I Lee, Jing-Long Huang, Li-Chen Chen, Kuo-Wei Yeh, Liang-Shiou Ou, Tsung-Chieh Yao, Chao-Yi Wu, Syh-Jae Lin

**Affiliations:** ^1^Division of Allergy, Asthma, and Rheumatology, Chang Gung University College of Medicine, Taoyuan, Taiwan; ^2^Department of Pediatrics, Chang Gung University College of Medicine, Taoyuan, Taiwan; ^3^Division of Pediatric Neurology, Chang Gung University College of Medicine, Taoyuan, Taiwan; ^4^Primary Immunodeficiency Care and Research (PICAR) Institute and Chang Gung Memorial Hospital, Chang Gung University College of Medicine, Taoyuan, Taiwan; ^5^Department of Pediatrics, New Taipei Municipal TuChen Hospital, New Taipei City, Taiwan

**Keywords:** X-linked agammaglobulinemia (XLA), Bruton's tyrosine kinase (*BTK*), contiguous gene deletion syndrome (CGS), *TIMM8A/DDP1* gene, deafness-dystonia-optic neuronopathy syndrome (DDON), Mohr-Tranebjaerg syndrome (MTS)

## Abstract

**Background:** X-linked agammaglobulinemia (XLA) is caused by a mutation of the Bruton's tyrosine kinase (*BTK*) gene and is the most common genetic mutation in patients with congenital agammaglobulinemia. The aim of this study was to analyze the clinical features, genetic defects, and/or *BTK* expression in patients suspected of having XLA who were referred from the Taiwan Foundation of Rare Disorders (TFRD).

**Methods:** Patients with recurrent bacterial infections in the first 2 years of life, serum IgG/A/M below 2 standard deviations of the normal range, and ≦2% CD19+B cells were enrolled during the period of 2004–2019. The frequency of infections, pathogens, B-lymphocyte subsets, and family pedigree were recorded. Peripheral blood samples were sent to our institute for *BTK* expression and genetic analysis.

**Results:** Nineteen (from 16 families) out of 29 patients had *BTK* mutations, including 7 missense mutations, 7 splicing mutations, 1 nonsense mutation, 2 huge deletions, and 2 nucleotide deletions. Six novel mutations were detected: c.504G>T [p.K168N], c.895-2A>G [p.Del K290 fs 23^*^], c.910T>G [p.F304V], c.1132T>C [p.T334H], c.1562A>T [p.D521V], and c.1957delG [Del p.D653 fs plus 45 a.a.]. All patients with *BTK* mutations had obviously decreased *BTK* expressions. *Pseudomonas* sepsis developed in 14 patients and led to both Shanghai fever and recurrent hemophagocytic lymphohistiocytosis (HLH). Recurrent sinopulmonary infections and bronchiectasis occurred in 11 patients. One patient died of *pseudomonas* sepsis and another died of hepatocellular carcinoma before receiving optimal treatment. Two patients with contiguous gene deletion syndrome (CGS) encompassing the *TIMM8A/DDP1* gene presented with early-onset progressive post-lingual sensorineural Deafness, gradual Dystonia, and Optic Neuronopathy syndrome (DDON) or Mohr-Tranebjaerg syndrome (MTS).

**Conclusion:** Pseudomonas sepsis was more common (74%) than recurrent sinopulmonary infections in Taiwanese XLA patients, and related to Shanghai fever and recurrent HLH, both of which were prevented by regular immunoglobulin infusions. Approximately 10% of patients belonged to CGS involving the *TIMM8A/DDP1* gene and presented with the DDON/MTS phenotype in need of aggressive psychomotor therapy.

## Introduction

X-linked agammaglobulinemia (XLA; OMIM 300300), first described in 1952 (1), represents the prototype of primary B cell deficiencies caused by mutations of the Bruton's tyrosine kinase (BTK) gene, a member of the Tec family of kinases localized on Xq21.3–Xq22, in the majority of male patients presenting with agammaglobulinemia (2). The XLA phenotype is characterized by a reduction or lack of mature B lymphocytes (≦2% of total lymphocytes), which is caused by a differentiation-transition blockage of B cell progenitors to mature B lymphocytes (3). Affected individuals have profound hypogammaglobulinemia and thus show increased susceptibility to bacterial infections, including sinusitis, otitis media, pneumonia, cellulitis, meningoencephalitis, gastroenteritis, and conjunctivitis (4). Onset usually occurs between 6 and 12 months of age, after consuming maternal transplacental IgG. The prognosis for individuals with XLA has markedly improved in the last 25 years as a result of earlier diagnosis, aggressive antibiotic therapy, and mainly through the use of replacement gamma globulin (intravenous or subcutaneous, IVIG or SCIG) to achieve an optimal serum IgG level (5, 6).

A total of 1,806 different *BTK* gene mutations are currently recorded in the Leiden Open Variation Database (https://databases.lovd.nl/shared/genes/BTK, last update on June 8, 2020). In accordance with the Rare Disease Control and Orphan Drug Act in Taiwan, patients who are suspected of having XLA are referred to our Primary Immunodeficiency Care and Research (PICAR) Institute for a molecular/genetic diagnosis and therapeutic suggestion. The aim of this study was to assess whether Taiwanese patients with XLA have unique manifestations and novel mutations.

## Materials and Methods

### Patients

Patients with an initial diagnosis of XLA according to the European Society for Immunodeficiencies (ESID) criteria were enrolled from 2004 to 2019 (6), including (1) male patients with recurrent bacterial infections in the first 2 years of life, (2) serum IgG/A/M below 2 standard deviations of the normal range for age, and (3) ≦2% CD19+B cells. After excluding secondary etiologies of proteinuria, protein losing enteropathy, malnutrition, and severe burns, a definitive diagnosis of XLA was then made if a *BTK* mutation was identified and/or there was an obvious decrease in *BTK* expression in monocytes (7).

The patients and the healthy controls provided written consent for data collection and publication of this study. All human samples were obtained under protocols approved by the Institutional Review Board at Chang Gung Memorial Hospital (protocol 201601893A3, and 104-9578A3) and met the Institutional Review Board standards for ethical conduct of research with human subjects in accordance with the Declaration of Helsinki.

### Molecular Analysis of the *BTK* Gene

Total RNA was isolated from peripheral blood mononuclear cells with TRIzol (Invitrogen, Carlsbad, CA). Reverse transcription of messenger RNA followed by polymerase chain reaction (PCR) were performed as previously described (8, 9). Two pairs of oligonucleotide primers were designed to cover the entire coding region of the *BTK* gene. BTK1: CAG TGT CTG CTG CGA TCG AG; BTKC1: CAG TGG AAG GTG CAT TCT TG (1,277 b.p.); BTK5: TCA TTG TCA GAG ACT CCA GC; BTKC2: TTG CTC AGA AGC CAC TAT CC (1,253 b.p.). If a specific mutation was identified, genomic DNA responsible for the candidate exon was amplified and confirmed again.

### Detection of *BTK* Gene Expressions

The expression of *BTK* in monocytes was evaluated by immunostaining from whole blood as previously described (7, 8). In brief, the red blood cells were removed using Lyse/Fix Buffer (BD Pharmingen, San Diego, CA), then incubated with CD14–PE (clone Mϕ, BD Pharmingen) for 20 min, permeabilized using Perm Buffer II (BD Pharmingen), and then stained with AlexaFluor647-conjugated anti-BTK Ab (clone53/BTK, BD Pharmingen) or isotype IgG2a (clone MOPC-173, BD Pharmingen) to identify epitopes between 2 and 172 amino acids of the Pleckstrin homology (PH) and Tec homology (TH) domains within the gating monocytes. The threshold of FSC was set as 5,000 and the data was analyzed by FlowJo 7.6 (Treestar, USA).

## Results

### Demographic and Clinical Features

Of the 29 male patients (from 26 unrelated families) included in this study, 19 (from 16 unrelated families) had *BTK* mutations (Table 1). The median age at onset of XLA in these 19 patients was 1.2 (range 0–16; mean 2.5 ± 3.6) years. The median delay between the first significant infection and IVIG infusion was 2.7 (range 0–16; mean 3.7 ± 4.1) years. The median age at diagnosis and in April 2020 were 5.0 (range 0.5–27; mean 6.2 ± 6.6) years and 16.0 (range 0.5–28; mean 15.0 ± 11.1) years, respectively. All of the patients received regular IVIG supplements for their hypogammaglobulinemia to decrease susceptibility to infections. Sepsis was the most common clinical feature (16/19, 84.2%) followed by recurrent sinopulmonary infections of sinusitis, otitis, and pneumonia (13/19, 68.4%), leading to bronchiectasis (in 11 patients). Nineteen sepsis episodes occurred in 16 patients (*pseudomonas* in 14 and *pneumococcus* sepsis in 5). Skin cellulitis and abscesses developed in 5 patients. Two patients had *campylobacter* colitis, 1 had *Salmonella* colitis, 1 had rotavirus enteritis, and 1 had arthropathy, but none had *Giardia lamblia*. Patient P9 suffered from recurrent hemophagocytic lymphohistiocytosis (HLH) before regular IVIG infusion. One patient died of *pseudomonas* sepsis at 6 months of age (P4), and one patient (P1-2) died due to hepatocellular carcinoma at 27 years before receiving IVIG infusion.

**Table 1:** Clinical features of the XLA patients with *BTK* genetic mutations by the referred year.

**Referred year**	**Onset/Dx/ current [death]**	**CD19%**	***BTK* expression % (>80%)**	Mutation/Exon (E)/Domain^*^	**Igs G/A/M/E mg/dL**	**Clinical manifestations (recurrent sinopulmonary infections, RSI; Bronchiectasis, B; Sepsis; Chronic diarrhea, CD), identified pathogens and eventful infections with family maternal carrier**	**New/ References**
2004 P1-1	1.5Y/5.3Y/19Y	0.2%	2.5%	c. 1562A>T; p.D521V; E15/Kinase	320/16/32/<7	CD, *Pseudomonas aeruginosa* sepsis, *Pseudomonas oryzihabitans* sepsis, meningitis, toxic megacolon, face lip gangrene	Novel
P1-2	16Y/27Y [HCC]	0.1%	1.8%		248/16/23/<7	RSI, B, *Streptococcus pneumonia* hip arthritis, *Staphylococcus aureus* cutaneous abscess, appendicitis, chronic hepatitis B and C, hepatomegaly, hepatocellular carcinoma; mortality of hepatocellular carcinoma	Novel
2004 P2	5.3Y/8Y/25Y	1.1%	0.5%	c. 1132T>C; p.T334H; E12/SH2	117/<6/27.3/250	RSI, B, *Pseudomonas aeruginosa* sepsis, *Streptococcus pneumonia* sepsis, *Staphylococcus aureus* cutaneous abscess	Novel
2004 P3-1	1.2Y/3Y/23Y	0.1%	0.4%	Int 14 (-2) A>G/Kinase c.1567-2A>G Del p.A523 fs 5^*^	106/<6/27.3/ <7	RSI, B, *Streptococcus pneumonia* sepsis, arthropath	(10)
P3-2	3Y/6Y/22Y	0.3%	1.5%		146/<6/27.3/25	RSI, B, *Streptococcus pneumonia* sepsis, *Campylobacter* colitis	
2004 P4	4M/6M [Sepsis]	1.3%	1.1%	Int 10 (-2)A>G/SH3 c.895-2A>G Del p.K290 fs 23^*^	<150/<23/<18/<7	*Pseudomonas aeruginosa* sepsis and mortality	Novel
2007 P5	9M/6Y/15Y	0.5%	2.2%	c.1921C>T; p.R641C/E19/Kinase	142/<23/<18/<7	*Pseudomonas aeruginosa*, sepsis, facial palsy	Novel
2008 P6	3Y/5Y/15Y	0.2%	0.2%	c.910T>G; p.F304V/E11/SH2	<150/<23/<18/<7	RSI, B, *Pseudomonas Aeruginosa* sepsis	(11)
2009 P7	2.1Y/8Y/19Y	0.8%	1.5%	c. 504 G>T p. K168N/E6/TH	201/<23/19/<7	RSI, B, *Streptococcus pneumonia* sepsis	Novel
2010 P8	8M/7Y/25Y	0.0%	2.1%	Int5(-2)A>G/PH c.392-2A>G p.V131G; Del 132-174 a.a.	<136/<24/<17/<7	RSI, B, empyema, failure to thrive	(12, 13)
2015 P9	1.2Y/4Y/8Y	0.2%	1.1%	Int 17(-1) G>A/Kinase c.1751-1G>A Del p.V452 fs 19^*^	113/<6/<27/<7	RSI, B, *Pseudomonas aeruginosa* sepsis, osteomyelitis, ecthyma gangrenosum, failure to thrive, HLH-like	(11, 14, 15)
2015 P10	4Y/20Y/28Y	0.5%	0.1%	Int8 (+2) T>C/TH2 c.776+2 T>C Del p.I197 fs 7^*^	346/<23/22/<7	RSI, B, *Pneumococcus* pericarditis, septic hip arthritis and sepsis, *Pseudomonas Aeruginosa* sepsis	(16)
2016 P11-1	6M/1Y/3Y	0.0%	0.2%	Del c.1957G; Del D653 fs plus 45 a.a./E19/Kinase	271/<23/14/<7	RSI, B, *Pseudomonas Aeruginosa* sepsis	Novel
2019 P11-2	0/1Y	0.2%	0.3%		212/< <23/<4/<7	Prenatal diagnosis	Novel
2017 P12	5M/6Y/16Y	1.0%	0.2%	c. 232 C>T; p.G78^*^/E3/PH	202/<46/5/<19	RSI, B, *Pseudomonas aeruginosa* sepsis, Ecthyma gangrenosum, Campylobacter enterocolitis and right foot cellulitis with abscess	(2, 17)
2018 P13	3.1Y/5Y/28Y	0.0%	2.1%	c.1385 G>A; p.G462D; E15/Kinase	22.3/<5.9/<17/<7	RSI, B, *Streptococcus pneumonia* sepsis, *Pseudomonas aeruginosa* sepsis, empyema, failure to thrive	(18)
2018 P14	2.2Y/3Y/5Y	0.0%	0.4%	Del E19 DDP1	213/<7/12/<7	RSI, otitis media, perianal cellulitis, *Pseudomonas Aeruginosa* sepsis dystonia	(19, 20)
2018 P15	1.2Y/1.5Y/3Y	0.1%	1.1%	Del E6-19 DDP1	209/<7/<18 <7	Rotavirus enteritis, *Pseudomonas aeruginosa* sepsis, ataxia, dystonia, hearing impairment	(19, 20)
2019 P16	6M/1Y/2Y	1.2%	1.5%	Int14 (+1) G>A/Kinase c.1349 +1 G>A Del p.W395 fs 32^*^	186/<23/22/<7	*Pseudomonas aeruginosa* sepsis (two times), *Salmonella* colitis	(21)

* TK, The tyrosine kinase domain; PH, Pleckstrin homology domain; SH2, Src homology 2 domain; SH3, Src homology 3 domain; TH, Tec homology domain; Del, deletion; fs, frameshift;

* *meant “stop” codon. The white and gray columns represented 16 unrelated families*.

One pair of nephews (P1-1, P1-2) and one pair of siblings (P3-1, P3-2) were diagnosed in 2004. Three of these patients (P1-2, P3-1, and P3-2) developed bronchiectasis, and one uncle (P1-2) died of hepatocellular carcinoma when his nephew (P1-1) suffered from *pseudomonas* sepsis. The third pair of siblings (P11-1, P11-2) were diagnosed in 2016 and 2019, respectively. The index case (P11-1) developed *pseudomonas* sepsis, and the younger (P11-2) was identified by prenatal genetic analysis and was free of significant infections under regular IVIG infusion.

### Genetic Analysis

**Figure 1:**
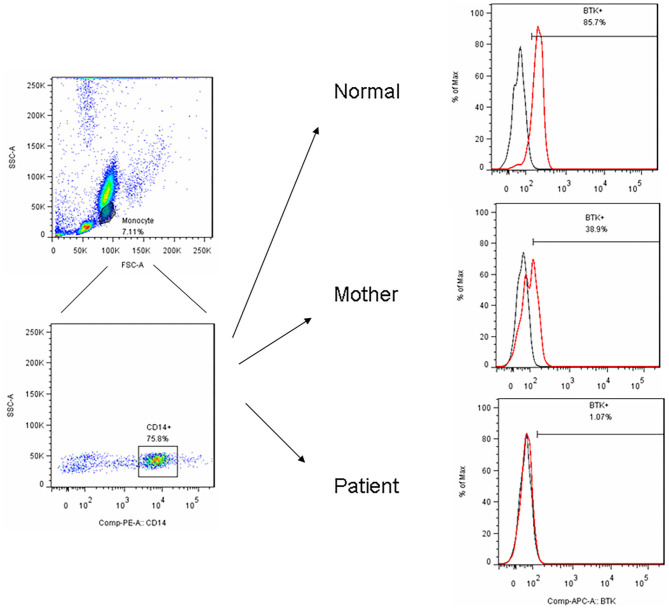
After PBMC purified by centrifugation, we utilized FSC and SSC to locate the monocyte region and gated them by CD14+ in a representative patient (P15) with the *BTK* mutations showed an almost complete absence of *BTK* expression (1.1%) and a bimodal pattern in his carrier mother (38.9%) compared to the normal healthy control (85.7%).

The whole coding region was sequenced using two pairs of primers for PCR-amplification cDNA that was reverse transcripted from RNA. If a variant was detected in cDNA PCR-amplification, the responsible exons with the flanking intronic regions of the *BTK* gene were again confirmed. Ten patients with a low CD19 cell count but normal *BTK* expression were, as expected, compatible with the wild type of the *BTK* gene (Supplemental Table 1). The *BTK* expression was almost absent in the 19 included patients (Figure 1), of whom 7 had missense (6 unrelated families) mutations, 7 had splicing (6 unrelated families) mutations, 4 had deletions (3 unrelated families, including 2 huge deletions), and 1 had a nonsense mutation (Table 1). The involved domains from the N-end to the C-end of the amino acid were the Pleckstrin homology domain (PH) in 2, the Tec homology domain (TH) in 1, Src homology 3 domain (SH3) in 1, Src homology 2 domain (SH2) in 2, tyrosine kinase domain (TK) in 11, and an additional 2 huge deletions affecting the last exon 19. The genetic defects were distributed through the whole coding region, and over half (11/19) were clustered in exons 14, 15, and 19. All of the mothers were carriers. Six unique mutations (in 8 patients) were identified (2, 10–21), including c.1562A>T, c.1132T>C, c.895-2A>G, c.504G>T, c.910T>G, and c.1957delG.

### Genotype–Phenotype Correlation

In contrast to other reports that patients with missense mutations had maintained some residual capability to therefore present with milder phenotypes than those with other types of mutation (7, 12, 13), all of our patients with missense mutations developed similar severity as sepsis and bronchiectasis to those with other mutation types. However, early initiation of IVIG seemed to attenuate the exacerbation of bronchiectasis and prevent sepsis episodes, because the patients who received regular IVIG before 2 years of age did not develop bronchiectasis.

**Figure 2:**
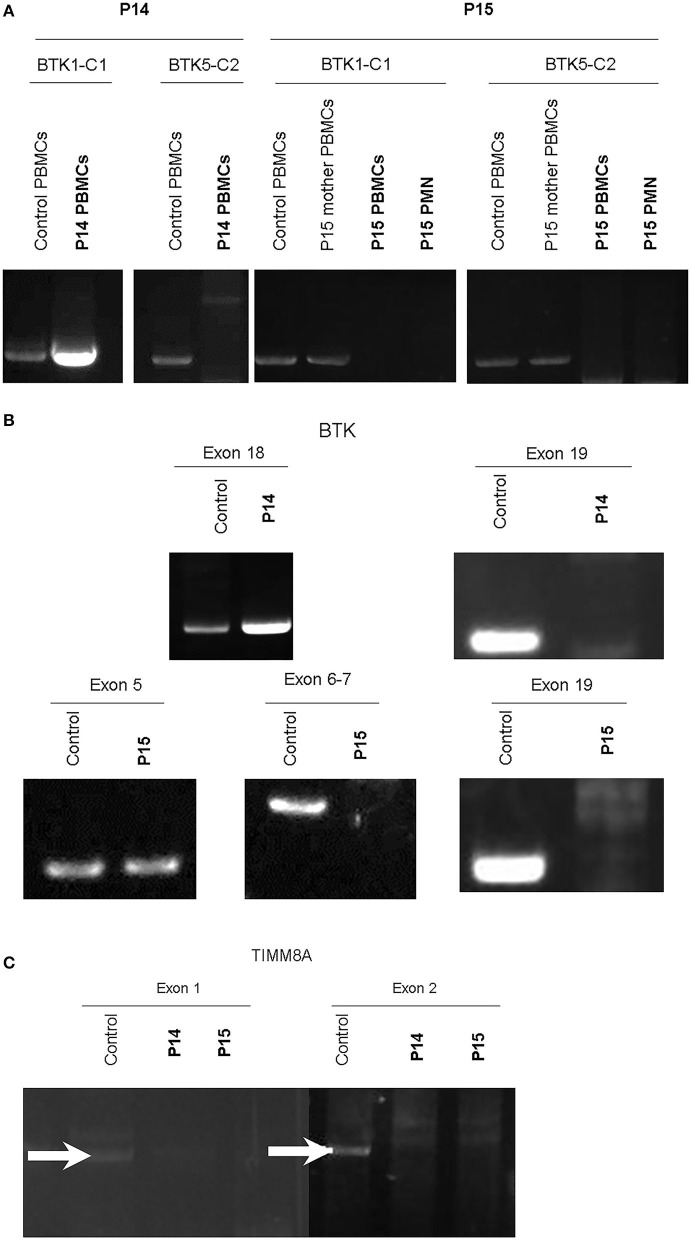
RT-PCR amplification of cDNA included two designed two pairs: BTK1-BTKC1 for the coding region from exon 1 to exon 13 (product 1,277 b.p.); and BTC5-BTKC2 (1,253 b.p.) for the coding region from exon 11 to exon 19. Compared to the healthy control and mother of P15, only cDNA amplification by RT-PCR of the product from BTK1-BTKC1 was right detectable in P14, and the others were all undetectable. PMNs should express *BTK*, but much lower than PBMCs. We evaluated *BTK* expression in two cell lines of PBMCs and PMNs in P15. The two leukocyte components did not express any *BTK* after PCR-amplification in P15 **(A)**. Therefore, each exon was amplified from genetic DNA. Exon 19 in the *BTK* gene in P14 and exons 6–19 in P15 were missing **(B)**. The contiguous gene TIMM8A with two exons was amplified, but it revealed only non-specific products in exon 1 (GGA GTT GGA CGC CTG CCT CGC; CTT GAA TCC TGT CAT GAT GAA for exon 1, product 1,593 b.p.) and undetectable in exon 2 (GAA CCT GGC GGA GGT TAC AGT; CCT TGG AAT CAG CCC ATG CTA, product 1,742 b.p.). In the normal control, two white-arrows pointed at the correct locations **(C)**. Two duplications were performed each.

**Figure 3:**
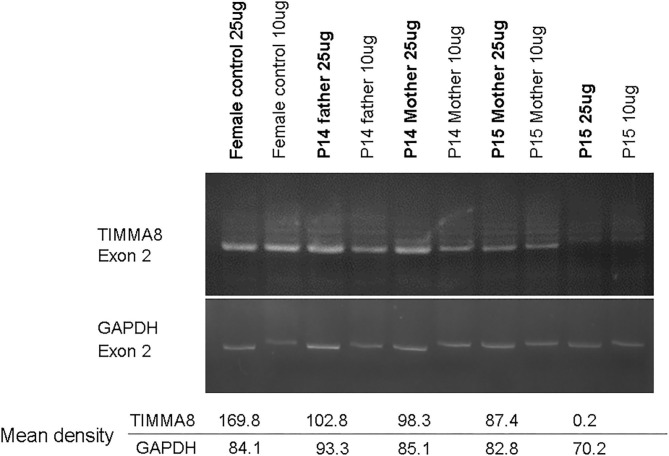
Under the same condition for PCR amplification of candidate genes to predict carrier status, the concentration-density ratio between TIMMA8-exon 2 (total 4 locus in two X chromosome) and GADPH-exon 2 (total 2 locus in one chromosome 12) was 198.8/84.1 in the normal non-carrier female. However, in these two carrier-mothers, the concentration-density between TIMMA8-exon 2 (total 2 locus in one X chromosome) and GADPH-exon 2 (total 2 locus in chromosome 12) was 98.3/85.1 in mother P14 and 87.4/82.8 in mother P15, and both were certainly carriers and equal to a male (P14 father with one X chromosome) 102.8/93.3. This implied the half-dose existence of the *TIMMA8* gene on X chromosome in their carrier-mothers compared to the normal healthy females.

Of note, in the 2 huge deletions, the designed PCR primers amplified the coding region of cDNA covering the segment of exons 1–13 in P14, but none were detected in P15 (Figure 2A). Furthermore, each exon from 1 to 19 was amplified, and the absence of exon 19 and the absence of exons 6–19 was in patients P14 and P15 (Figure 2B), who both gradually developed dystonia and ataxia. The *TIMM8A/DDP1* (Translocase of Inner Mitochondrial Membrane 8A/Deafness-Dystonia Peptide 1) gene is 770 b.p. downstream from the 3′ end of the *BTK* gene in the X chromosome, and it encodes DDP1 which imports metabolite transporters from the cytoplasm to mitochondria and mainly orchestrates neural development and muscle coordination. Contiguous gene deletion syndrome (CGS) affected the *BTK* and *TIMM8A* genes supported by the evidence of undetectable two exons of the *TIMM8A* gene (Figure 2C). Compared to a normal female with two X alleles, two carrier-mothers of P14 and P15 had only half the relative concentration of exon 2 in the *TIMM8A* gene (Figure 3) and exon 19 of the *BTK* gene (data not shown) that was equal to a healthy male with one X allele.

## Discussion

Referred from the Taiwan Foundation of Rare Disorders (TFRD), 19 of 29 male patients (from 26 unrelated families) with hypogammaglobulinemia, low B cell populations, and infections were diagnosed with XLA. The prevalence of XLA based on approximately 3,200,000 live births during the 16-year study period (https://www.ncbi.nlm.nih.gov/books/NBK448170/) is estimated around 1 case per 170,000 live births in Taiwan, close to that of Norway (1/100,000–1/285,000) (22) and Switzerland (1/200,000) (23), but higher than Italy (1/250,000) (24) and USA (1/379,000) (4). As well as geno-geographic diversity for higher prevalence, our national health insurance (NHI) and medicine strategy covering such rare disorders encourage affected patients to urge effective management for life-quality improvement.

As expected, obviously decreased intracellular *BTK* staining in monocytes prompts analysis of genetic defects in those with lower B cell percentage. Eight patients (8/19, 42%) had novel mutations, and two (2/19, 10.5%) had huge deletions involving the neighboring *TIMM8A* gene encoding mitochondrial import inner membrane translocase subunit TIMM8A that is expressed at much higher levels in Purkinje cells of the cerebellum (19). Thus, patients with CGS develop deafness-dystonia-optic neuronopathy syndrome (DDON) [or Mohr-Tranebjaerg syndrome (MTS)] characterized by progressive dystonia, ataxia, hearing impairment, cortical blindness, and early dementia. However, XLA patients present with hearing impairment which is more commonly ascribed to complications of recurrent sinopulmonary infections, and those with unstable gait are often suspected of having chronic encephalitis caused by enterovirus or prion (John Cunningham) infections. Both neurological dysfunctions of hearing impairment and unstable gait are often thought to be due to B cell deficiencies rather than neuronopathy in auditory brain stem responses caused by the loss of TIMM8A in mitochondrial dysfunction. Thus, it is important for clinicians to consider the possibility of a deletion of the last exon 19 in the *BTK* gene in patients suspected of having DDON syndrome, and to further test for the existence of the neighboring *TIMM8A* gene to allow for an effective therapeutic strategy. Applying to Italian, Chinese, American, and African cohort studies (4, 12, 24, 25), those with huge deletions of the last exon 19 in the *BTK* gene should have, but not yet, further evaluated the existence of *TIMM8A* gene for the DDON/MTS phenotype.

Conversely, exon 1 of the *TIMM8A* gene follows exon 19 of the *BTK* gene. If patients with the DDON phenotype are identified to have a deletion of exon 1 of the *TIMM8A* gene, the diminished *BTK* expression in flow cytometry and/or a B cell percentage <2% are considered to be clues to speculate whether the deletion expands to the *BTK* gene, thereby allowing for timely IVIG treatment. Overall, of 19 patients identified with CGS in a Medline search (19, 20, 26–32) including our patients P14 and P15, only one of five with a deletion of the last exon 19 in the *BTK* gene and the whole *TIMM8A* gene succumbed to pneumonitis and respiratory failure because of rapid progressive severe spasticity at 6 years of age. In contrast, the other four who had larger deletions expanding from exon 6 in the *BTK* gene seemed to have gradual psychomotor retardation, speech impairment, and sensorineural hearing loss. Their phenotypic severity did not correlate to the extent of the deletion (20).

Under NHI coverage and support by social welfare, Taiwanese infants are obligated to receive regular vaccination and evaluation of neurodevelopment. If they have cough and yellow rhinorrhea over 5 days in suspicion of sinusitis or otitis media, primary physicians often give empiric antibiotics (Augmentin or cefaclor). Our vaccine schedules of *Haemophilus influenzae* type B (Hib) and pneumococcus 13-valent conjugate vaccine are both at 2 and 4 months old (plus the third dose of pneumococcus 13-valent conjugate vaccine at 6 months old) and therefore decrease Hib and pneumococcus infections in all children, including those with *BTK* mutations. Our previous sepsis study in PIDs patients showed that the most identified pathogen was *pseudomonas* followed by *strep. pneumococcus* (33, 34). Until now, there have been no available pseudomonas vaccines to prevent pseudomonas infections. These aspects may explain how pseudomonas sepsis has become the most common presentation in patients with *BTK* mutations. Thus, our patient P1-1 had a [D521V] *BTK* missense mutation and presented with the Shanghai fever phenotype consisting of severe bloody diarrhea, neutropenia, eczema gangrenosum, and pseudomonas sepsis (35). Because the 10 warning signs proposed by the Jeffery Model Foundation were not widely known in Taiwan in 2004, patient P4 died of pseudomonas sepsis when he was 5 months old despite receiving the first IVIG infusion at that time. Patient P1-2, the maternal uncle of P1-1, died of hepatocellular carcinoma with chronic hepatitis B and C at 29 years of age. Recently, our thalassemia patient (manuscript preparation) received three doses of anti-CD20 deletion therapy (rituximab 375 mg/m^2^ per week for 3 doses) for post-transplant autoimmune pancytopenia. Rituximab continuously inhibited B cell generation until now, unexpectedly over 6 years, and led to persistent hypogammaglobulinemia. The precise mechanism of malignant transformation remains elusive despite higher virus load of hepatitis B virus in hypogammaglobinemia patients than those without hypogammaglobinemia. Hepatitis B vaccine can enhance memory B cells to make high-affinity antibody to effectively resist against hepatitis B virus and therefore may prevent hepatocellular carcinoma (36). However, hypogammaglobinemia patients (with *BTK* mutations or CD20 deletion by biologics) are presumed not to produce enough high-affinity antibodies to the hepatitis B virus. Whether such insufficiency antibodies for neutralization and opsonization to hepatitis B virus in hypogammaglobinemia patients relate to malignancy transformation should be further investigated.

Recurrent hemophagocytic lymphohistiocytosis (HLH) occurred before regular IVIG infusions in our 4-year-old patient P9 who suffered from pseudomonas sepsis or/and recurrent sinopulmonary infections, which could trigger HLH. The mechanism of hypercytokine storm from HLH resembling macrophage activation syndrome infers that the absence of *BTK* can augment inflammation cytokines through Toll-like receptor signaling pathways (TLR4, 7, 8, and 9) (37–39), possibly driving to the HLH process as two brothers in a previous report (40). IVIG infusion serves as an induction medication in the TPOG-2004-HS protocol for HLH to suppress hypercytokinemia and therefore modulate the overactive innate immunity related to the *BTK* mutations.

This study should be interpreted in light of its limitations. First, our patients had hypogammaglobinemia, lower B cell percentage, and typical antibody-deficiency phenotypes. However, additional two Taiwanese male siblings with the p.P116L *BTK* mutation had selective IgM deficiency and focal proliferative glomerulonephritis initially presenting with proteinuria and hematuria (41). Both <1% B cells reminded physicians of XLA despite a normal IgG level. Thus, male patients with a low B cell percentage and increased susceptibility to bacterial infections should be screened for the *BTK* mutation. Second, to those with the DDON phenotype accompanying recurrent otitis media, sinusitis, or/and pneumonia, we recommend assessing their *BTK* expression level and investigating whether they belong to CGS involving the *BTK* and *TIMM8A* genes. Third, although carrier detection can be indirectly predicted by half-dose of X-linked targeted genes compared to non-carrier females or a bimodal flowcytometric pattern of the *BTK* expression in CD14+monocytes, the direct breakpoints at genomic DNA should be possibly located by designed “walking” primers with/without ligation-medicated PCR (28, 42) through ligation-adapter as well as whole genome sequencing (WGS) that could explore the un-amplified sequence alignment by bioinformatics software program. Fourth, genetic mutations of non-X linked agammaglobulinemia encoding for pre-BCR and/or BCR complex (such as *IGHM, CD79a, CD79b*, and *IGLL1* genes) and for activating mTOR signaling (such as *PI3KD* and *PIK3R1* genes). These candidate genes and others are investigated in referred patients with the wild *BTK* gene by whole exome sequencing (some patients in Supplemental Table 1), but negative findings (43).

In conclusion, regular IVIG infusions and adequate prophylactics prevented recurrent sinopulmonary infections in Taiwanese patients with *BTK* mutations except two who died due to hepatocellular carcinoma and pseudomonas sepsis before IgG infusion in 2004. Pseudomonas sepsis was the most common manifestation, and these patients also presented with severe diarrhea and eczema gangrenosum, relating to Shanghai fever and recurrent HLH. In addition to the higher rate of novel mutations of the *BTK* gene (42% in this study), approximately 10% of patients were CGS affecting the *BTK* and *TIMM8A* genes and presented with the DDON/MTS phenotype characterized by early-onset progressive post-lingual sensorineural deafness, gradual dystonia, and optic atrophy, which indeed required aggressive psychomotor re-education and physical therapy.

## Data Availability Statement

The raw data supporting the conclusions of this article will be made available by the authors, without undue reservation.

## Ethics Statement

The studies involving human participants were reviewed and all human samples were obtained under protocols approved by the Institutional Review Board at Chang Gung Memorial Hospital (protocol 201601893A3 and 104-9578A3) and met the Institutional Review Board standards for ethical conduct of research with human subjects in accordance with the Declaration of Helsinki. Written informed consent to participate in this study was provided by the participants' legal guardian/next of kin.

## Author Contributions

Y-HY, M-YH, and W-IL carried out the molecular genetic studies, analyzed the sequence alignment, and drafted the manuscript. S-JL and C-YW performed the immunoassays. W-IL designed the study and the genetic analysis. L-CC, K-WY, T-CY, C-YW, L-SO, and J-LH participated in the study to care for critical patients. All authors read and approved the final manuscript.

## Conflict of Interest

The authors declare that the research was conducted in the absence of any commercial or financial relationships that could be construed as a potential conflict of interest.
