# Supplementary material for: Distinct Clinical Features and Novel Mutations in Taiwanese Patients With X-Linked Agammaglobulinemia
Source: Front Immunol. 2020 Sep 4;11:2001. doi: 10.3389/fimmu.2020.02001 (PMC7498534; doi:10.3389/fimmu.2020.02001)
Supplement: Supplementary file 1 [file Table_1.DOC]

Supplemental Table 1. Clinical features of the patients with hypogammaglobulinemia but normal *BTK* gene

| **Referred year** | **Onset/ Dx/ Current** | **CD19%** (>5%) | ***BTK***  **Expression**  (>80%) | **Mutation/**  **Exon (E)** | **Igs**  **G/A/M/E** | **Clinical manifestations (Recurrent sinopulmonary infections, RSI; Bronchiectasis, B; Sepsis; Chronic diarrhea, CD), identified pathogens and eventful infections** |
| --- | --- | --- | --- | --- | --- | --- |
| 2003  H1 | 12Y/ 18Y/ 37Y | 3% | 89% | Wild | 372/12/<4/<7 | RSI, B, |
| 2007  H2 | 15Y/ 21Y/ 34Y | 2.1% | 92% | Wild | 225<23<32/<7 | RSI, B |
| 2009  H3 | 9Y/ 20Y/ 31Y | 2.2% | 98% | wild | 271/<23/<4/<17 | RSI, B, sepsis |
| 2010  H4 | 51Y/ 62Y/ 72Y | 2.2% | 89% | Wild | 435/35/23/96 | Severe pneumonia after thymoma |
| 2015  H5 | 1D/ 4Y/ 9Y | 3.2% | 89% | Wild | 324/<23/45/<7 | Prematurity, sepsis |
| 2015  H6 | 15Y/22Y/ 27Y | 2.1% | 92% | Wild | 453/<23/24/15 | *Pseudomonas aeruginosa* sepsis, Erythema gangrenosum |
| 2017  H7 | 1Y./ 3Y/ 6Y | 2.4% | 89% | Wild | 225<23<32/<7 | Prematurity, RSI |
| 2017  H8 | 18Y/ 26Y/ 29Y | 2.2% | 91% | Wild | 436<24/<34/127 | RSI, B |
| 2019  H9-1 | 4M/ 1Y/ 2Y | 2.2% | 92% | Wild | 202/<23/5/<19 | prematurity |
| 2019  H9-2 | 4M/ 2Y/ 3Y | 3.5% | 89% | Wild | 202/<23/5/<19 | prematurity |
